# Human occupation, not forest structure, determines sand fly abundance in the Amazon

**DOI:** 10.1186/s13071-026-07409-x

**Published:** 2026-04-30

**Authors:** Andreia Fernandes Brilhante, Fredy Galvis-Ovallos, Felipe Trovalim Jordão, Leandro José Ramos, Roberto Cardoso Ilacqua, Wandercleyson Uchôa Abreu, Melissa Suzanne Nolan, Fernanda Portela Madeira, Glauco Martins Silva, Thais Costa Santos, Paula Ribeiro Prist, Denis Valle, Marcia Aparecida Sperança, Gabriel Zorello Laporta

**Affiliations:** 1 Center for Health Sciences and Sports, Federal University of Acre, Rio Branco, AC Brazil; 2 Department of Epidemiology, School of Public Health, University of São Paulo, São Paulo, SP Brazil; 3 Center for Natural and Human Sciences, Federal University of ABC, São Bernardo do Campo, SP Brazil; 4 Graduate Program in Health Sciences, FMABC University Center, Santo André, SP Brazil; 5 Department of Epidemiology and Biostatistics, Arnold School of Public Health, University of South Carolina, Columbia, SC USA; 6 Institute for Infectious Disease Translational Research, University of South Carolina, Columbia, SC USA; 7 Multidisciplinary Center, Forest Campus, Federal University of Acre, Cruzeiro do Sul, AC Brazil; 8 Forests and Grasslands Programme, IUCN, Washington, DC USA; 9 School of Forest, Fisheries, and Geomatics Sciences, University of Florida, Gainesville, FL USA

**Keywords:** Amazon region, Bayesian analysis, Deforestation, Disease vectors, Land use, *Leishmaniasis*, Cutaneous, Psychodidae, Spatial analysis

## Abstract

**Background:**

Across Amazonian deforestation frontiers, phlebotomine sand flies transmit *Leishmania* spp., the causative agents of American cutaneous leishmaniasis (ACL). Landscape modification can alter vector ecology and transmission risk, yet the relative roles of forest cover, landscape configuration, and deforestation timeline remain poorly understood. We evaluated how landscape composition and configuration, assessed across multiple spatial scales, and deforestation timeline influence sand fly abundance and *Leishmania* infection in Cruzeiro do Sul, Acre, Brazil.

**Methods:**

Sand flies were collected at 20 study sites during two cross-sectional surveys conducted in 2022 and 2024. Landscape metrics, including forest cover and edge density, were quantified within circular buffers of 3, 5, and 7 km^2^ around each site. Deforestation timeline, defined as time since initial forest loss, was used as a proxy for the duration of human occupation. Associations with sand fly abundance and *Leishmania* infection were assessed using Bayesian regression models, applying negative binomial models for abundance and binomial models for infection probability. Infection was detected using quantitative polymerase chain reaction (PCR) and confirmed by Sanger sequencing.

**Results:**

Forest cover and edge density, across all spatial scales, were not associated with sand fly abundance or *Leishmania* infection. In contrast, longer deforestation timelines were consistently associated with higher sand fly abundance, driven largely by increased captures of the sand fly species *Nyssomyia antunesi*. No landscape variable showed a clear association with infection occurrence.

**Conclusions:**

Sand fly abundance in this Amazonian frontier was associated with the duration of human occupation rather than with current forest structure. These findings suggest that vector populations can persist in human-modified landscapes and highlight the importance of incorporating deforestation timeline into ACL surveillance and risk assessment.

**Graphical Abstract:**

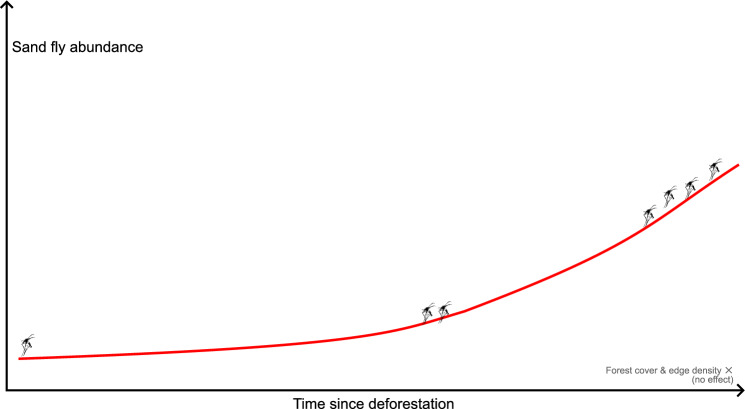

**Supplementary Information:**

The online version contains supplementary material available at 10.1186/s13071-026-07409-x.

## Background

Phlebotomine sand flies are the primary vectors of *Leishmania* spp., the causative agents of American cutaneous leishmaniasis (ACL) in humans across the Brazilian Amazon [1–4]. ACL transmission is ecologically complex, involving multiple reservoir hosts, sand fly vectors, and *Leishmania* species interacting within heterogeneous landscapes [1, 2, 5].

Habitat alteration and deforestation have long been proposed as drivers of ACL transmission through their effects on vector and host communities. Nevertheless, ACL is traditionally regarded as a zoonosis largely associated with relatively preserved forest ecosystems [3, 6, 7], where forest integrity is assumed to sustain sylvatic transmission cycles. Under this framework, landscape composition (forest cover) and configuration (fragmentation and edge density) are expected to influence vector abundance and infection patterns relevant to ACL transmission.

Evidence from several Amazonian settings, however, shows that some sand fly species persist in disturbed or human-modified environments. In the Marajó Archipelago (Pará state) *Evandromyia walkeri*, *Evandromyia infraspinosa*, and *Nyssomyia antunesi* have been recorded in periurban settings, demonstrating persistence in disturbed landscapes [2]. Similarly, in Presidente Figueiredo (Amazonas state), disturbed habitats supported rodent communities dominated by generalist species with higher *Leishmania* infection rates [5]. In Acre state, human ACL cases have been reported in urbanized areas adjacent to forest fragments, where sand fly species such as *Ny*. *antunesi*, *Ny*. *whitmani*, and *Ev*. *walkeri* have been captured in domestic and peridomestic environments [8–10]. These observations suggest that vector–host interactions relevant to ACL transmission may extend beyond intact forest settings under conditions of human disturbance.

Beyond the structural characteristics of the landscape, the temporal dimension of anthropogenic disturbance may also influence sand fly populations and infection dynamics. This temporal dimension, referred to here as the deforestation timeline, reflects the time elapsed since initial forest loss and human settlement at a given site [11, 12]. While cumulative deforestation and long-term land-use change have been associated with ACL incidence in the Amazon [3], the relative contribution of deforestation timeline, as distinct from static measures of landscape composition and configuration, remains unclear.

In this study, we tested the hypothesis that sand fly abundance and *Leishmania* infection rates would be higher in landscapes characterized by greater forest cover and lower levels of forest fragmentation, as predicted by classical models of sylvatic transmission of ACL. We conducted an observational study across 20 sites in Cruzeiro do Sul, Acre state, a deforestation frontier in the Brazilian Amazon, selected to represent contrasting social and environmental contexts. At each site, sand flies were captured and assessed for *Leishmania* infection status.

By examining associations between vector abundance, infection status, landscape composition and configuration across multiple spatial scales, and deforestation timelines, this study builds on the multifunctional landscape concept [4]. The findings are intended to inform ecological countermeasures, that is, landscape-based strategies for vector control that integrate disease prevention with spatial planning, land management, and conservation objectives.

## Methods

### Study area and rationale

**Fig. 1:**
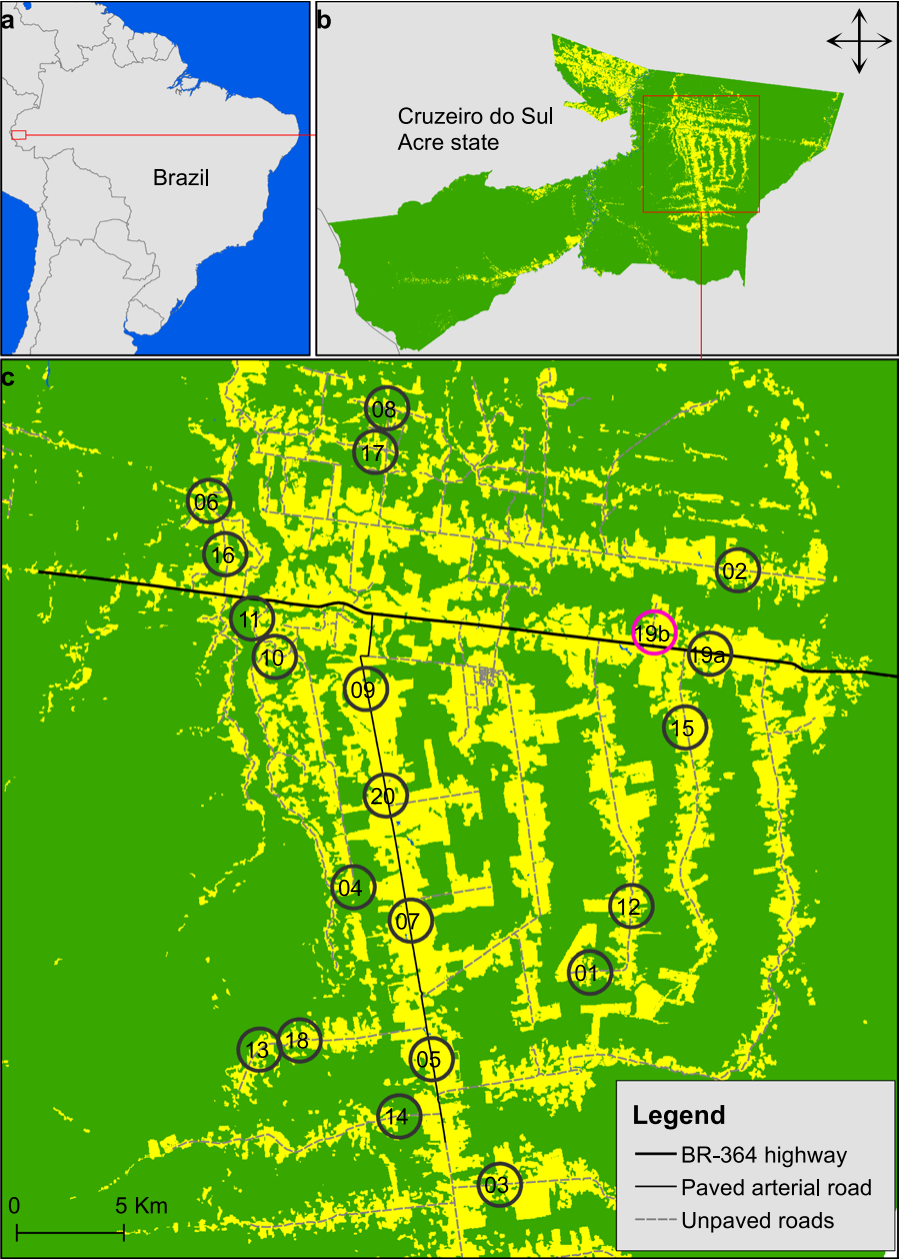
Land-use and land-cover map of Santa Luzia rural settlement, Cruzeiro do Sul, Acre state, Brazil, in 2022 (**a**, **b**). Green areas represent forest remnants, while yellow areas represent anthropogenic habitats (pasture for cattle and secondary vegetation) (**c**). Black circles with numbers indicate the 20 study sites selected for the first cross-sectional survey conducted in 2022, distributed across landscapes with contrasting forest cover, configuration, and deforestation timeline (**c**). The pink circle indicates the replacement of site 19, which became inaccessible in 2024 (**c**)

The study was conducted in the Santa Luzia rural settlement, municipality of Cruzeiro do Sul, located in the westernmost region of Acre state, Brazil (Fig. 1). Unlike regions such as Pará or Rondônia states, where deforestation has occurred over decades and contributed to high leishmaniasis endemicity, deforestation in Cruzeiro do Sul is comparatively recent and unevenly distributed in space [13–15]. The average annual incidence rate of ACL in this municipality is around 3 cases per 10,000 inhabitants, which is considered lower than the rates observed in the more deforested eastern parts of Acre state [13, 16, 17]. The primary risk profile for *Leishmania* infection is the forest/sylvatic cycle of ACL, which mainly affects young male workers in rural areas [16]. This epidemiological context allows assessment of whether sand fly abundance and *Leishmania* infection are associated with relatively preserved forest habitats, as traditionally proposed, or with landscapes shaped by recent deforestation and land-use change.

By following study sites that differed in forest cover, landscape configuration, and deforestation timeline, we assessed how frontier-related landscape transformation is associated with key transmission parameters, including sand fly abundance and infection rates. This approach allowed us to evaluate whether patterns described in long-established Amazonian frontiers are also observed in regions with more recent human occupation.

### Study site selection and landscape characterization

A total of 20 study sites were selected across the municipality of Cruzeiro do Sul, Acre state, Brazil (Fig. 1) [18], between June and July 2022. Each site was defined by a central sand fly sampling point and its surrounding landscape, characterized within fixed-area circular buffers (see below). Site selection combined spatial patterns of deforestation with predictive modeling outputs [12].

Selection criteria included: (i) the proportion of native forest cover derived from Sentinel-2 imagery acquired in 2022, (ii) deforestation timeline, defined as time since initial forest loss, (iii) proximity to primary healthcare facilities as a proxy for human accessibility, and (iv) projected deforestation probability for 2025 estimated using a random forest model and deforestation data from 2010 to 2020 [12]. These criteria ensured representation of sites spanning contrasting stages of frontier occupation and landscape transformation.

All sites were georeferenced in July 2022 using a GPS + GLONASS receiver (Garmin International, Olathe, KS, USA) under the UTM WGS 1984 Zone 19S projection. During the second survey conducted in 2024, site 19a became inaccessible owing to land access restrictions and was replaced by site 19b (pink circle in Fig. 1), which exhibited comparable forest cover, landscape configuration, and deforestation timeline.

High-resolution multispectral Sentinel-2 images (10 m spatial resolution) from August 2022 and July 2024 were obtained from the Copernicus Open Access Hub (European Space Agency, Paris, France). After atmospheric correction and preprocessing, supervised land-use and land-cover classification was conducted using the Semi-Automatic Classification Plugin in QGIS version 3.22.7 [12].

To quantify landscape composition and configuration, classified maps were analyzed using Fragstats version 4.2.1. Landscape composition was expressed as the proportion of native forest cover, while landscape configuration was quantified as the density of native forest edge. These metrics were calculated within circular landscapes of 3, 5, and 7 km^2^ surrounding each sampling site [11].

The selection of these spatial extents was guided by ecological considerations, reflecting the reported dispersal capacity of phlebotomine sand flies, thereby capturing landscape features potentially influencing vector movement and habitat use beyond the immediate sampling point [19, 20].

Deforestation timeline was calculated as the number of years elapsed from the onset of deforestation to the year of sampling (2022 or 2024). The onset of deforestation was defined as the year in which cumulative native forest loss first exceeded 10% within the 7 km^2^ landscape surrounding each site. For sites with settlement histories extending back to the 1970s, Landsat 1–9 imagery (USGS, Reston, VA, USA) from 1975 to 2024 was analyzed to identify the earliest detectable forest loss. Sites for which deforestation occurred prior to the availability of satellite imagery were assigned a minimum deforestation timeline of > 47 years, corresponding to forest loss occurring before 1975. This timeline-based approach is described in detail elsewhere [21].

Each site corresponds to a rural settlement lot where families cultivate cassava and maintain small pastures. The region is characterized by tropical moist forest, gentle topography (200–300 m), dystrophic yellow argisol soils, and a high water table that ensures year-round water availability [22, 23]. These environmental and land-use attributes reflect the forest–agriculture mosaic typical of early stage deforestation frontiers in the southwestern Amazon.

### Sampling procedures

Sand fly collections were carried out at the 20 study sites during two cross-sectional surveys conducted in July 2022 and July 2024, ensuring comparable seasonal conditions in both surveys. Sampling was restricted to peridomestic environments and the adjacent forest edge at each site.

At each site, three standardized trapping approaches were employed: (i) a Centers for Disease Control and Prevention (CDC) light trap installed near the house, (ii) a Shannon trap operated by one or two collectors at the house–forest edge transition, and (iii) a second CDC light trap placed at the forest edge. Traps operated concurrently between 17:00 and 22:00.

The three trapping approaches were implemented simultaneously at each site and treated as subreplicates representing the peridomestic–forest interface, rather than as independent sampling units for positional comparisons. For this reason, sand fly captures were pooled by site and survey year for analysis linking vector abundance and infection to landscape attributes.

In July 2022, females were killed with ethyl acetate and dissected in sterile saline to examine the digestive tract for flagellates and the spermathecae for species identification. In July 2024, females were similarly killed, their genitalia exposed for identification, and specimens preserved in 80% ethanol at −20 °C for subsequent molecular analyses. Males collected in both years, as well as a subset of females from each survey, were cleared following the method of Forattini [19] and identified using the taxonomic key by Galati [20]. Despite dissections in 2022, all female specimens from both surveys were later analyzed by polymerase chain reaction (PCR) for *Leishmania* DNA, with positive amplifications detected only in 2024.

### Detection of *Leishmania* DNA

*Leishmania* DNA was investigated in female sand flies collected in 2024. Total DNA was extracted following proteinase K digestion and organic extraction [24], and screened using a TaqMan quantitative PCR (qPCR) assay targeting the single-copy chitinase (*CHIT*) gene to detect and differentiate *Leishmania* species complexes [25]. Samples positive by qPCR were re-amplified by conventional PCR for Sanger sequencing, and species identification was further confirmed by amplification and sequencing of the *ITS1* rDNA region [26]. Positive and negative controls were included in all reactions [27–29]. Sequence data were deposited in GenBank (accession no. PX530579). Methodological details are provided in Additional File 1: Supplementary Text S1, and positive samples are listed in Additional File 2: Supplementary Table S1.

### Hypothesis testing

We evaluated the relationships between three response variables: (i) sand fly abundance in 2022, (ii) sand fly abundance in 2024, and (iii) the number of *Leishmania*-infected sand flies in 2024. We tested the hypothesis that these outcomes were associated with (i) landscape composition and configuration, represented by the proportion and edge density of remaining native forest cover, and (ii) the deforestation timeline, defined as the number of years since deforestation began. Landscape metrics were calculated at multiple spatial extents (3, 5, and 7 km^2^ landscapes) to account for the dispersal capacity of phlebotomine sand flies.

Sand fly abundance at each site (VAi) was modeled as following a negative binomial distribution to account for overdispersion in count data:

$$VA_{i} \sim NegBinom\left( {\mu_{i} ,s} \right)$$

$$\mu_{i} = \exp \left( {x_{i}^{T} \beta^{VA} } \right)$$

where ${\mu }_{i}$ is the mean, $s$ is the dispersion parameter, ${x}_{i}^{T}$ is the design vector of independent variables, and ${\beta }^{VA}$ is the vector of regression coefficients.

The number of *Leishmania*-infected sand flies ($I{V}_{i}$) was modeled using a binomial distribution conditional on total sand fly abundance:

$$IV_{i} \sim Binom\left( {VA_{i} ,p_{i}^{IV} } \right)$$

$$p_{i}^{IV} = \frac{{\exp \left( {x_{i}^{T} \beta^{IV} } \right)}}{{1 + \exp \left( {x_{i}^{T} \beta^{IV} } \right)}}$$

where ${\beta }^{IV}$ represents the regression coefficients for infection probability.

Although sand fly abundance was measured at the same sites in 2022 and 2024, we analyzed each survey year separately to reflect the cross-sectional nature of each survey, and this allowed us to assess whether associations between landscape attributes and sand fly abundance were consistent across independent sampling periods. Evidence for robustness was inferred when similar patterns were observed in both years.

All models were estimated under a Bayesian framework to avoid reliance on asymptotic assumptions and to allow regularization through prior specification. Weakly informative priors were assigned to model parameters: intercepts followed $N\left(\mathrm{0,10}\right)$ distributions, slope coefficients followed $N\left(\mathrm{0,1}\right)$ distributions, and the negative binomial dispersion parameter ($s$) was assigned a uniform prior between 0 and 1000.

As a sensitivity analysis, we fit an alternative model to assess whether the effect of forest loss varied with time since deforestation. In this model, sand fly abundance was regressed on accumulated deforestation (defined as 100% minus the percentage of remaining forest cover), deforestation timeline, and their interaction. All covariates were standardized prior to analysis. This analysis was conducted as a sensitivity test to evaluate whether sand fly abundance was higher in landscapes combining extensive forest loss with longer histories of human occupation. Results are presented in Additional File 3: Supplementary Table S2.

## Results

After 600 h of standardized sampling effort (200 h with CDC traps and 100 h with Shannon traps per survey period), we captured 161 sand flies in July 2022 (73 females and 88 males) and 453 sand flies in July 2024 (267 females and 186 males), corresponding to mean capture rates of 0.5 and 1.5 specimens per hour, respectively. Sand fly abundance was therefore approximately three times higher in 2024 than in 2022. The most abundant species included taxa with confirmed or putative vector status for ACL in Acre state (Table 1).

**Table 1:** Frequency of sand flies by species, sex, collection method, and vector status across 20 study sites in the study area, Cruzeiro do Sul, Acre, during July 2022 and July 2024

Species^a^	July 2022			July 2024			Vector status^b^
	*N* (%)	F:M	Method	*N* (%)	F:M	Method	
*Ev*. *saulensis*	2 (1)	2:0	Shannon	1 (0.5)	1:0	Shannon	–
*Ev*. *walkeri*	12 (7)	1:11	Shannon	18 (4)	12:6	Shannon, CDC	–
*Lu*. *evangelistai*	0 (0)	–	–	1 (0.5)	1:0	CDC	–
*Lu*. *gomezi*	1 (1)	1:0	Shannon	0 (0)	–	–	–
*Lu*. *sherlocki*	0 (0)	–	–	4 (1)	3:1	Shannon, CDC	–
*Mi*. *micropyga*	0 (0)	–	–	1 (0.5)	1:0	CDC	–
*Ny*. *antunesi*	115 (71)	46:69	Shannon, CDC	346 (76)	197:149	Shannon, CDC	Putative vector of ACL agents [30]
*Ny*. *fraihai*	5 (3)	5:0	Shannon, CDC	1 (0.5)	1:0	CDC	–
*Nyssomyia* sp.	0 (0)	–	–	1 (0.5)	1:0	CDC	–
*Pa*. *abonnenci*	0 (0)	–	–	2 (0.5)	1:1	CDC	–
*Pa*. *bigeniculata*	0 (0)	–	–	1 (0.5)	1:0	CDC	–
*Pa*. *dendrophyla*^c^	2 (1)	2:0	Shannon	2 (0.5)	2:0	CDC	–
*Pa*. *punctigeniculata*	1 (1)	1:0	Shannon	0 (0)	–	–	–
*Pi*. *nevesi*	1 (1)	1:0	CDC	0 (0)	–	–	–
*Ps*. *amazonensis*	2 (1)	2:0	CDC	0 (0)	–	–	–
*Ps*. *davisi*	20 (13)	10:0	Shannon, CDC	71 (15)	48:23	Shannon, CDC	Putative vector of ACL agents [30]
*Th*. *ruifreitasi*	0 (0)	–	–	4 (1)	0:4	Shannon, CDC	–

^a^*Ev.* *Evandromyia*, *Lu.* *Lutzomyia*, *Mi.* *Micropygomyia*, *Ny.* *Nyssomyia*, *Pa.* *Psathyromyia*, *Pi.* *Pintomyia*, *Ps.* *Psychodopygus*, *Th.* *Trichophoromyia*

^b^Potential vector status with supporting references

^c^A female specimen identified with flagellates and nematodes following dissection. *F* female, *M* male

*Nyssomyia antunesi* dominated collections in both surveys, accounting for 71% of captures in 2022 and 76% in 2024, followed by *Psychodopygus davisi* (Table 1). These species are recognized or suspected vectors of ACL agents in the region. Additionally, a single female specimen of *Psathyromyia dendrophyla* was microscopically identified carrying flagellates and nematodes (Additional File 4: Supplementary Video S1). This specimen was collected at one of the most deforested sites (site 5; Table 2).

**Table 2:** Female sand fly counts and landscape variables for the 20 study sites, including forest cover, edge density at three spatial scales, and deforestation timeline, Santa Luzia rural settlement, 2022

Site	Females in 2022	Forest cover 3 km^2^ (%) 2022	Forest cover 5 km^2^ (%) 2022	Forest cover 7 km^2^ (%) 2022	Edge density 3 km^2^ (m/ha) 2022	Edge density 5 km^2^ (m/ha) 2022	Edge density 7 km^2^ (m/ha) 2022	Deforestation timeline (years) 2022
1	1	52	58	60	96	73	70	6
2	0	26	39	45	80	78	73	27
3	1	32	34	34	96	94	94	23
4	8	65	67	65	125	116	114	18
5	2^a^	11	17	25	22	36	45	35
6	4	46	51	55	191	196	202	22
7	9	4	11	19	37	51	54	38
8	1	60	58	61	180	182	197	5
9	0	12	13	14	34	37	41	36
10	0	29	35	39	86	92	107	37
11	33	60	54	52	86	89	99	47
12	6	34	45	52	115	99	88	14
13	1	54	60	63	164	175	189	15
14	0	65	65	64	80	77	72	15
15	1	25	35	45	85	94	89	27
16	1	49	47	44	73	66	62	27
17	0	55	60	63	98	103	107	8
18	3	45	59	63	101	91	86	28
19a	1	14	17	26	58	59	68	47
20	1	19	22	25	88	77	69	38

^a^*Pa*. *dendrophyla* female found with flagellates and nematodes (Additional File 4: Supplementary Video S1)

Landscape composition and configuration varied markedly among the 20 study sites (Table 2). Forest cover ranged from 4% to 65% within 3 km^2^ landscapes, while edge density showed similarly wide variation across spatial extents (3, 5, and 7 km^2^). Deforestation timelines spanned from 5 to more than 47 years, reflecting heterogeneous histories of land occupation across the settlement.

Female sand fly abundance in 2022 showed substantial spatial heterogeneity, ranging from 0 to 33 females per site, with site 11 exhibiting the highest capture (Table 2).

In July 2024, *Leishmania* DNA was detected in sand flies from four sites (sites 10, 11, 12, and 15; Table 3). Site 11 again yielded the highest number of sand flies, with 175 females collected, suggesting temporal stability in relative site-level abundance between survey years. At this site, 11 sand flies were infected, including 10 *Ny*. *antunesi* and 1 *Ev*. *walkeri*. Ten sequences matched *Leishmania amazonensis* or closely related taxa, and one corresponded to *Leishmania* (*Viannia*) spp. (Additional File 2: Supplementary Table S1).

**Table 3:** Sand fly infection status, female counts, and landscape metrics at the 20 study sites, Santa Luzia rural settlement, 2024

Site	Infected^a^ in 2024	Females in 2024	Forest cover 3 km^2^ (%) 2024	Forest cover 5 km^2^ (%) 2024	Forest cover 7 km^2^ (%) 2024	Edge density 3 km^2^ (m/ha) 2024	Edge density 5 km^2^ (m/ha) 2024	Edge density 7 km^2^ (m/ha) 2024	Deforestation timeline (years) 2024
1	0	1	47	53	54	91	80	83	8
2	0	0	29	42	49	103	96	86	29
3	0	4	36	38	37	116	107	102	25
4	0	0	27	70	67	100	72	77	20
5	0	15	12	18	27	26	37	43	37
6	0	25	41	48	53	112	120	110	24
7	0	1	6	15	23	66	82	81	40
8	0	3	62	59	60	85	84	83	7
9	0	2	19	36	22	63	166	71	38
10	1	15	28	33	39	99	99	109	39
11	11	175	63	57	55	86	91	101	49
12	1	5	38	48	54	111	94	83	16
13	0	0	54	61	62	61	59	56	17
14	0	1	64	64	63	94	97	95	17
15	1	11	25	37	47	88	93	87	29
16	0	0	48	45	42	73	64	57	29
17	0	0	39	47	53	74	76	71	10
18	0	7	35	51	57	115	112	103	30
19b	0	2	26	32	38	87	88	95	40
20	0	0	19	22	25	86	81	76	40

^a^Additional details on the infected sand flies are provided in Additional File 2: Supplementary Table S1

Additional infections with *Leishmania amazonensis*-like parasites were detected in *Ps*. *davisi* at sites 10 and 15, and in *Nyssomyia* sp. at site 12 (Additional File 2: Supplementary Table S1). No infected sand flies were detected at the remaining sites.

Most sites exhibited relatively modest changes in forest cover between 2022 and 2024, consistent with the gradual, mosaic-like progression of deforestation in Amazonian rural settlements. Nevertheless, changes were scale-dependent. Some sites experienced localized forest loss (e.g., sites 17 and 18 lost 16% and 10% of forest cover within 3 km^2^ landscapes, respectively), whereas others showed gains attributable to natural regeneration (e.g., site 9 gained 7% forest cover within 3 km^2^) (Tables 2 and 3).

This scale dependence is illustrated by site 4, which showed a 38% reduction in forest cover at the 3 km^2^ scale, while forest cover increased by 2–3% at the 5 and 7 km^2^ scales. Such patterns suggest that localized deforestation can be offset by broader-scale forest recovery, possibly reflecting land-use shifts in which cattle ranching moves from older degraded plots to newly cleared areas, allowing natural regeneration. Overall, these short-term changes did not substantially alter the rank order of sites in terms of forest cover or edge density.

Table 4 presents posterior mean regression coefficients with their 95% credible intervals and Bayesian *P*-values, calculated as $(p\left(\beta <0\right),p(\beta >0))$, where $p()$ denotes the posterior probability. Because no single spatial extent consistently outperformed others on the basis of model deviance, results are shown for the 7 km^2^ landscapes, which integrate a broader-scale landscape context.

**Table 4:** Bayesian regression slope estimates describing associations between sand fly abundance and *Leishmania* infection with landscape variables measured within a 7 km^2^ landscape

Landscape variables	Abundance 2022 Posterior mean (95% credible interval), Bayesian *P*-values	Abundance 2024 Posterior mean (95% credible interval), Bayesian *P*-values	Infection 2024 Posterior mean (95% credible interval), Bayesian *P*-values
Forest cover (7 km^2^)	0.50 (−0.24 to 1.24), *P* = 0.09	0.35 (−0.70 to 1.29), *P* = 0.22	0.40 (−0.73 to 1.62), *P* = 0.26
Edge density (7 km^2^)	0.12 (−0.60 to 0.91), *P* = 0.37	0.33 (−0.36 to 0.99), *P* = 0.17	0.07 (−082 to 1.09), *P* = 0.46
Deforestation timeline	0.84 (0.23–1.48), *P* = 0.006*	1.09 (0.42–1.79), *P* = 0.004*	0.25 (−0.37 to 0.98), *P* = 0.25

Sand fly abundance was assessed in July 2022 and July 2024, whereas *Leishmania* infection was assessed only in July 2024

^*^Posterior probabilities indicate strong evidence for a positive association (Bayesian *P* < 0.05)

Forest cover and edge density showed no clear associations with sand fly abundance in either 2022 or 2024, and no associations with *Leishmania* infection probability (Table 4). In contrast, deforestation timeline was positively associated with sand fly abundance in both years, with strong posterior support (2022: posterior mean = 0.84, *P* = 0.006; 2024: posterior mean = 1.09, *P* = 0.004). No landscape variable showed a clear association with infection occurrence, although the number of detected infections was limited.

These findings contrast with our original expectations, as higher forest cover and lower edge density, evaluated across multiple spatial extents (3–7 km^2^), would typically be hypothesized to be associated with higher sand fly abundance or infection under classical sylvatic transmission models of ACL. However, such relationships were not observed (Table 4).

**Fig. 2:**
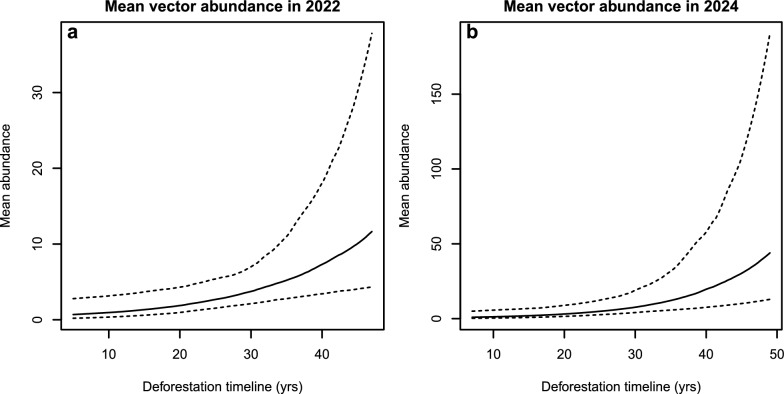
Predicted sand fly abundance (with 95% credible intervals) across the deforestation timeline (years since first deforestation) for 2022 (**a**) and 2024 (**b**)

Figure 2 shows the predicted relationship between sand fly abundance and deforestation timeline. Across both survey years, longer deforestation timelines were consistently associated with higher sand fly abundance, suggesting temporal stability in this relationship despite interannual variation in overall capture rates. Forest cover and edge density showed no meaningful effects across the modeled range.

Results from the sensitivity analysis are presented in Additional File 3: Supplementary Table S2. When sand fly abundance was modeled as a function of accumulated deforestation, deforestation timeline, and their interaction, no consistent evidence was found for an effect of accumulated deforestation or for an interaction between accumulated deforestation and deforestation timeline in either sampling year. In contrast, the deforestation timeline remained positively associated with sand fly abundance, with posterior support in 2022 and weaker evidence in 2024. These findings suggest that sand fly abundance was not preferentially associated with landscapes combining extensive forest loss and longer deforestation timelines but was more consistently associated with the deforestation timeline. This pattern is consistent with the main results of the primary analysis.

## Discussion

This study tested whether sand fly abundance and infection would be higher in landscapes with more continuous forest and greater habitat integrity [19]. However, our results did not support this expectation. Forest cover and edge density were not associated with either sand fly abundance or *Leishmania* infection. Instead, sand fly abundance was consistently and positively associated with the deforestation timeline, and this pattern was consistent across both sampling years. Abundance and species composition remained similar between 2022 and 2024, suggesting persistence of local sand fly communities once established.

*Leishmania* infections showed no clear association with any landscape variable, which may be consistent with parasite circulation in both recently disturbed and long-established deforested areas. However, this result should be interpreted with caution given the limited number of detected infections. Natural infection rates in Amazonian sand fly populations are typically low (~1–10%) [31], and the number of positives observed here falls within this range. Nevertheless, the small sample size reduces statistical power to detect associations with landscape variables, and the absence of detectable effects does not imply the absence of an ecological relationship.

In models including both accumulated deforestation and its interaction with timeline, only the timeline term was associated with sand fly abundance, reinforcing the interpretation that long-term human occupation may be more strongly linked to vector abundance than the extent of forest removal per se.

Overall, these findings indicate substantial ecological flexibility of Amazonian sand fly populations in human-modified landscapes and question the assumption that relatively intact forest environments are required to sustain ACL transmission.

The sand fly composition and diversity observed here align with expectations for Acre state, a region known for high sand fly biodiversity and elevated ACL risk [30, 32–36]. *Nyssomyia antunesi*, the dominant species across both sampling periods, is a putative ACL vector known to tolerate degraded environments and to occur frequently in close association with humans and domestic animals [2, 30]. Even at relatively low densities, ACL transmission risk can remain high, depending on the host community [5]. In Rio Branco, the capital of Acre, *Ny*. *antunesi* may serve as an important vector for *Leishmania*, as it occurs in high population densities, has been detected carrying *Leishmania* DNA, and feeds predominantly on humans [30, 31, 33, 34].

Our study provides, to our knowledge, the first confirmed detection of *L*. *amazonensis* DNA in *Ny*. *antunesi* supported by concordant molecular assays. Previous vector studies in Acre have predominantly reported circulation of *Leishmania* (*Viannia*) spp.; however, *Leishmania* (*Leishmania*) parasites, likely *L*. *amazonensis*, have been detected in sand flies from forested areas in Xapuri, including *Brumptomyia* spp., *Ny*. *shawi*, *Ps*. *lainsoni*, and *Ps*. *llanosmartinisi* [31]. These findings, together with evidence from other Amazonian studies, suggest that *Ny*. *antunesi* can harbor multiple *Leishmania* species and persist under diverse environmental pressures [37–41]. Further investigation into its vector competence, feeding behavior, and ecological plasticity is therefore warranted.

The second most abundant species in our collections, *Ps*. *davisi*, is an anthropophilic sand fly widely distributed across the Amazon and frequently associated with deforested landscapes and areas affected by infrastructure development [31, 32, 35, 42–44]. This species has repeatedly been detected with *L*. *braziliensis* DNA and exhibits diverse blood-meal sources, reinforcing its epidemiological relevance in altered environments [31, 32, 35, 44, 45]. The co-occurrence of *Ny*. *antunesi* and *Ps*. *davisi* at high densities across sites with varying degrees of disturbance supports the notion that these vectors are well adapted to the heterogeneous landscapes typical of Amazonian settlement frontiers.

*Evandromyia walkeri* also deserves attention. Although not formally incriminated as a vector, this species has a broad geographic distribution, has been detected carrying different *Leishmania* species, and is frequently associated with anthropized forest environments [30, 33, 34, 46, 47]. Its detection with *Leishmania* DNA in our study suggests that it may participate opportunistically in local transmission cycles, particularly in modified landscapes.

Sand flies capable of transmitting ACL may also harbor other pathogens. For example, *Psychodopygus carrerai* in Acre has been reported carrying nematodes of the family Onchocercidae, although this finding requires further confirmation [48]. Similarly, in our study, one specimen of *Pa*. *dendrophyla* collected on a degraded site carried worm-like organisms and flagellates (Supplementary Video S1), showing the broader parasitological diversity associated with sand flies in frontier landscapes.

Overall, our results indicate that sand fly abundance is more strongly linked to long-term human occupation than to contemporary forest cover or edge density. Rather than being constrained by current landscape configuration, sand fly populations appear to establish and expand gradually as settlements stabilize and human activities persist over time. This finding suggests that historical land-use trajectories may be more informative for understanding vector dynamics than static measures of forest structure [49]. Landscape management therefore remains relevant, because long-term land-use decisions shape the ecological context in which synanthropic vector populations become established [4]. This interpretation is consistent with previous studies showing that sand fly species associated with ACL transmission can persist in peridomestic and anthropized environments across the Amazon [50].

Despite this overall pattern, substantial heterogeneity was observed among sites with similar occupation histories. This variability suggests that additional ecological processes operating at finer spatial scales may influence local vector populations, beyond the landscape metrics captured in this study. It is important to note that these patterns were observed during the dry season, when both environmental disturbance and sand fly population structure may differ from wetter periods [31].

One plausible but biologically consistent explanation is that sand fly populations in these frontier landscapes operate under metapopulation-like dynamics, in which local populations undergo cycles of extinction and recolonization across a mosaic of habitat patches. In this framework, peridomestic environments may function as key population sources for anthropophilic and disturbance-tolerant species such as *Nyssomyia* spp. and *Psychodopygus* spp. The peridomestic environment may represent a critical ecological unit sustaining local population persistence and recolonization dynamics. These species are frequently reported in human-modified and peridomestic settings across the Amazon [2, 30, 32–34, 37, 38]. Evidence from Rondônia and other Amazonian regions indicates that several vector species persist and maintain infection across both forest and peridomestic environments, showing their ecological capacity to exploit anthropogenic habitats [32, 37, 38, 43, 44].

In our study system, each sampling site corresponded to an inhabited rural lot, where peridomestic conditions are shaped by long-term human activities. These environments typically include domestic animal husbandry, accumulation of organic matter (e.g., cassava residues), and shaded and humid soil conditions. Together, these factors create suitable microhabitats for sand fly breeding, resting, and host-seeking behavior in disturbed and peridomestic habitats [2, 50, 51].

Importantly, the relationship between deforestation timeline and sand fly abundance did not appear strictly linear. Visual inspection of model predictions (Fig. 2), together with the increasing slope of the predicted response, suggests an acceleration in abundance after approximately three decades of occupation, consistent with a potential threshold effect. Although this pattern should be interpreted cautiously given the observational design and uncertainty intervals, it raises the hypothesis that sand fly populations may respond nonlinearly to the cumulative effects of human settlement. Under this interpretation, the deforestation timeline acts not only as a proxy for time since forest loss but also for the progressive establishment and maturation of peridomestic conditions that enhance habitat suitability for vectors [50, 51].

At the same time, the observed variability in sand fly abundance among sites with similar deforestation timelines likely reflects limitations in our proxy for colonization history. The timeline was estimated retrospectively using satellite imagery, based on the year in which forest loss exceeded 10% within a 7 km^2^ landscape. While this approach provides a standardized and reproducible metric [21], it does not capture variation in the intensity, continuity, or quality of human occupation and land use. Differences in household structure, land management practices, animal husbandry, and environmental sanitation may therefore contribute to the heterogeneous patterns observed across sites, even under similar timelines of deforestation. This limitation may partially explain the heterogeneity observed among sites with comparable estimated timelines.

Currently, no ideal vaccines or fully effective drugs provide long-term protection or complete parasite clearance for ACL [52]. Vector control is also limited, especially for exophagic species that bite outdoors. While insecticide-treated nets can reduce indoor transmission, they do little to prevent bites from outdoor sand flies [53, 54].

In this context, our findings suggest that ACL risk management should explicitly account for the long-term ecological consequences of human settlement and land-use history, particularly the progressive establishment of peridomestic environments that sustain vector populations [4, 12]. Interventions targeting peridomestic conditions (improved environmental management, reduction of organic matter accumulation, and management of domestic animal shelters) may represent more effective and context-specific strategies in frontier settings.

Broader landscape-based or incentive-driven conservation strategies (e.g., payments for ecosystem services) may still offer co-benefits for ecosystem services and climate resilience, but their role in directly reducing ACL transmission remains uncertain and requires further empirical evaluation [12, 55–57].

Single-day collections at each site yielded relatively low sand fly counts but were sufficient to reveal consistent patterns in abundance and species composition across sampling periods. However, limited sampling duration may have reduced the detection of short-term fluctuations or rare species.

Molecular detection of *Leishmania* was constrained by assay sensitivity. The *CHIT* TaqMan qPCR outperformed conventional *ITS1* PCR, and several *CHIT*-positive samples failed to amplify the *ITS1* fragment [58], likely owing to low parasite loads. These limitations should be considered when interpreting infection estimates.

Sampling was restricted to a single month (July) during the dry season, a period in which forest disturbance is typically more intense and sand fly populations may be skewed toward older, more infectious individuals. Seasonal variation in abundance and species composition is well documented, and site-specific dominance patterns may shift throughout the year. Although dominant taxa such as *Nyssomyia* spp. often persist across seasons [31], our design does not capture seasonal turnover, and extrapolation beyond the sampling period should be made with caution.

Finally, because sampling was restricted to peridomestic environments, our design does not allow direct comparison with fully forested (sylvatic) sites.

## Conclusions

Sand fly abundance in this Amazonian deforestation frontier was more strongly associated with the duration of human occupation than with current forest cover or landscape configuration. These findings indicate that long-term land-use history is a key determinant of vector presence and persistence in ACL transmission settings. By showing the role of settlement-driven ecological change, our results suggest that ACL risk in frontier landscapes is shaped less by remaining forest structure than by the cumulative effects of human-modified environments over time. Incorporating land-use trajectories into surveillance and risk assessment frameworks may improve the targeting of control strategies in endemic regions.

## Supplementary Information

Additional file 1: Text S1. Detection and molecular characterization of *Leishmania* DNA.

Additional file 2: Table S1. Molecular detection and identification of *Leishmania* DNA in sand flies using *CHIT* TaqMan qPCR and *ITS1* conventional PCR. *CHIT*-positive samples were sequenced using the same primer set, and resulting fragments were compared with reference sequences in GenBank. *ITS1* amplification and sequencing were performed only for *CHIT*-positive specimens.

Additional file 3: Table S2. Sensitivity analysis of sand fly abundance in relation to accumulated deforestation, deforestation timeline, and their interaction, estimated separately for July 2022 and July 2024.

Additional file 4: Video S1. Nematodes located in or near the Malpighian tubules, and *Leishmania*-like parasites observed in the midgut of a female sand fly (*Pa*. *dendrophyla*) under light microscopy (400x magnification).

## Data Availability

All data are available in the figures, tables, and supplementary material.
