# Supplementary material for: Human occupation, not forest structure, determines sand fly abundance in the Amazon
Source: Parasit Vectors. 2026 Apr 30;19:225. doi: 10.1186/s13071-026-07409-x (PMC13192195; doi:10.1186/s13071-026-07409-x)
Supplement: Supplementary file 1 — Additional file 1: Text S1. Detection and molecular characterization of Leishmania DNA. [file 13071_2026_7409_MOESM1_ESM.docx]

**Additional file 1: Text S1** Detection and molecular characterization of *Leishmania* DNA

Female sand flies collected in 2024 were lysed in proteinase K buffer and incubated at 56 °C for 16 h. Total nucleic acids were extracted using a Trizol-type reagent following the manufacturer’s recommendations and resuspended in 10 mM Tris-HCl (pH 7.5). DNA integrity was verified on 1% agarose gels.

Screening for *Leishmania* DNA was performed using a TaqMan quantitative PCR (qPCR) assay targeting a conserved variable region of the single-copy chitinase (*CHIT*) gene. Amplification employed primers Quit792InfDoF (5’-GACCGGACCACACC-3’) and Quit933R (5’-TTGCGGCGGTGGCTG-3’), designed based on a published protocol. Three group-specific hydrolysis probes were used to discriminate *Leishmania* complexes:

- Viannia_FAM 5’–CGAGGCTACCTACAACAAGC–3’ (*Leishmania* (*Viannia*) spp.)
- MexAm_HEX 5’–CGAGGGTATATACAACAGGCGTC–3’ (*L*. *amazonensis*/*L*. *mexicana* group)
- Infdo_TexasRed 5’–GAGGCTGCATACAACAGGCG–3’ (*L*. *infantum* / *L*. *donovani* complex)

Reactions (20 µL) contained GoTaq^®^ Probe qPCR Master Mix, primers, probes, and nuclease-free water, and were run on a CFX96 Real-Time PCR System under the cycling conditions: 94°C for 3 min; 40 cycles of 94°C for 30 s, 64°C for 30 s, and 72°C for 30 s, with fluorescence acquisition at 64°C for 30 s.

Positive controls included DNA from reference strains of *L*. *amazonensis* (MHOM/BR/73/M2269), *L*. *braziliensis* (MHOM/BR/75/M2903), and *L*. *infantum* (MHOM/BR/74/PP/75). Negative controls consisted of DNA from non-*Leishmania* organisms (insects, humans, dogs, and *Trypanosoma cruzi*) and no-template controls.

Samples positive by qPCR were re-amplified using the same *CHIT* primers in a conventional PCR to generate amplicons for Sanger sequencing. In addition, a fragment of the internal transcribed spacer 1 (*ITS1*) rDNA was amplified using primers LITSR and L5.8S. PCR products were visualized on agarose gels, purified, and sequenced by the Sanger method. Sequence identity was confirmed by comparison with reference sequences, and the *ITS1* sequence generated in this study was deposited in GenBank (accession PX530579). Individual qPCR-positive samples are listed in Additional file 2: Table S1.
