# Supplementary material for: Human occupation, not forest structure, determines sand fly abundance in the Amazon
Source: Parasit Vectors. 2026 Apr 30;19:225. doi: 10.1186/s13071-026-07409-x (PMC13192195; doi:10.1186/s13071-026-07409-x)
Supplement: Supplementary file 2 — Additional file 2: Table S1. Molecular detection and identification of Leishmania DNA in sand flies using CHIT TaqMan qPCR and ITS1 conventional PCR. CHIT-positive samples were sequenced using the same primer set, and resulting fragments were compared with reference sequences in GenBank. ITS1 amplification and sequencing were performed only for CHIT-positive specimens. [file 13071_2026_7409_MOESM2_ESM.docx]

**Additional file 2: Table S1** Molecular detection and identification of *Leishmania* DNA in sand flies using *CHIT* TaqMan qPCR and *ITS1* conventional PCR. *CHIT*-positive samples were sequenced using the same primer set, and resulting fragments were compared with reference sequences in GenBank. *ITS1* amplification and sequencing were performed only for *CHIT*-positive specimens

| **Specimen** | **Site** | | **Capture method** | **Sand fly species** | ***CHIT* (chitinase) qPCR (amplicon)^a,b^** | ***Leishmania* species (*CHIT*)^c^** | **Top BLAST hit (*CHIT* fragment)** | ***ITS1* (rDNA) conventional PCR** | ***ITS1* GenBank accession (this work)** | ***Leishmania* species (*ITS1*)^3^** |
| --- | --- | --- | --- | --- | --- | --- | --- | --- | --- | --- |
| 5 | 11 | Shannon trap (house–forest interface) | | *Nyssomyia antunesi* | Positive | *L*. *amazonensis* | 100% to AY518257.1 | — | — | — |
| 7 | 11 | Shannon trap (house–forest interface) | | *Nyssomyia antunesi* | Positive | *Viannia* spp. (*L*. *braziliensis*) | 100% to OY748410.1 | — | — | — |
| 9 | 11 | Shannon trap (house–forest interface) | | *Nyssomyia antunesi* | Positive | *L*. *amazonensis* | 100% to AY518257.1 | — | — | — |
| 16 | 11 | Shannon trap (house–forest interface) | | *Nyssomyia antunesi* | Positive | *L*. *amazonensis* | 100% to AY518257.1 | — | — | — |
| 17 | 11 | Shannon trap (house–forest interface) | | *Nyssomyia antunesi* | Positive | *L*. *amazonensis* | 100% to AY518257.1 | — | — | — |
| 95 | 11 | Shannon trap (house–forest interface) | | *Nyssomyia antunesi* | Positive | *L*. *amazonensis* | 100% to AY518257.1 | — | — | — |
| 107 | 11 | Shannon trap (house–forest interface) | | *Nyssomyia antunesi* | Positive | *L*. *amazonensis* | 100% to AY518257.1 | — | — | — |
| 118 | 11 | Shannon trap (house–forest interface) | | *Nyssomyia antunesi* | Positive | *L*. *amazonensis* | 100% to AY518257.1 | — | — | — |
| 129 | 11 | Shannon trap (house–forest interface) | | *Nyssomyia antunesi* | Positive | *L*. *amazonensis* | 100% to AY518257.1 | — | — | — |
| 152 | 11 | Shannon trap (house–forest interface) | | *Nyssomyia antunesi* | Positive | *L*. *amazonensis* | 100% to AY518257.1 | Positive | PX530579 | *L*. *amazonensis* |
| 165 | 11 | Shannon trap (house–forest interface) | | *Evandromyia walkeri* | Positive | *L*. *amazonensis* | 100% to AY518257.1 | — | — | — |
| 175 | 15 | CDC trap (near house) | | *Psychodopygus davisi* | Positive | *L*. *amazonensis* | 100% to AY518257.1 | — | — | — |
| 204 | 10 | CDC trap (near house) | | *Psychodopygus davisi* | Positive | *L*. *amazonensis* | 100% to AY518257.1 | — | — | — |
| 232 | 12 | CDC trap (near house) | | *Nyssomyia* sp. | Positive | *L*. *amazonensis* | 100% to AY518257.1 | — | — | — |

**^a^**The *CHIT* TaqMan qPCR assay was more sensitive than the *ITS1* conventional PCR; therefore, several *CHIT*-positive samples did not amplify the *ITS1* fragment, likely due to low parasite loads in individual sand flies.

**^b^***CHIT* amplicons (<100 bp) could not be deposited in GenBank because the database does not accept sequences shorter than 100 bp.

**^c^**Species assignments for both markers are based on the closest BLAST match.
