# Supplementary material for: Human occupation, not forest structure, determines sand fly abundance in the Amazon
Source: Parasit Vectors. 2026 Apr 30;19:225. doi: 10.1186/s13071-026-07409-x (PMC13192195; doi:10.1186/s13071-026-07409-x)
Supplement: Supplementary file 3 — Additional file 3: Table S2. Sensitivity analysis of sand fly abundance in relation to accumulated deforestation, deforestation timeline, and their interaction, estimated separately for July 2022 and July 2024. [file 13071_2026_7409_MOESM3_ESM.docx]

**Additional file 3: Table S2** Sensitivity analysis of sand fly abundance in relation to accumulated deforestation, deforestation timeline, and their interaction, estimated separately for July 2022 and July 2024

| **Covariate** | **Posterior mean (2022)** | **95% credible interval (2022)** | **Bayesian *P*-value (2022)** | **Posterior mean (2024)** | **95% credible interval (2024)** | **Bayesian *P*-value (2024)** |
| --- | --- | --- | --- | --- | --- | --- |
| Accumulated deforestation (AcDef) | −0.39 | −1.16 to 0.43 | 0.17 | −0.15 | −1.14 to 0.88 | 0.39 |
| Deforestation timeline | 0.72 | −0.06 to 1.50 | **0.03*** | 0.68 | −0.19 to 1.53 | 0.07 |
| AcDef × Timeline | −0.34 | −1.24 to 0.57 | 0.23 | −0.88 | −1.87 to 0.24 | 0.06 |

*Indicates posterior evidence suggestive of a positive association (Bayesian *P* < 0.05).
